# Sarcopenia Abdominal Muscle Mass Index Assessment Informs Surgical Decision-Making in Displaced Fractures of the Femoral Neck

**DOI:** 10.3390/jcm14082573

**Published:** 2025-04-09

**Authors:** Filip Brzeszczyński, David Hamilton, Angela Dziedzic, Marek Synder, Oktawiusz Bończak

**Affiliations:** 1 Department of Trauma, Orthopaedics and Musculoskeletal Oncology, Copernicus Memorial Hospital, Pabianicka 62, 93-513 Łódź, Poland; 2 Research Centre for Health, Glasgow Caledonian University, Govan Mbeki Building, Cowcaddens Road, Glasgow G4 0BA, UK; 3 Department of General Biochemistry, Faculty of Biology and Environmental Protection, University of Lodz, ul. Pomorska 141/143, 90-236 Łódź, Poland; 4 Orthopaedic and Paediatric Orthopaedics Department, Medical University of Lodz, Pomorska 251, 92-213 Łódź, Poland

**Keywords:** fractures of the femoral neck, sarcopenia, muscle mass, postoperative outcomes

## Abstract

**Background**: Displaced femoral neck fractures (FNFs) can be treated with hemiarthroplasty (HA) or total hip arthroplasty (THA), with THA typically offered to fitter patients. Sarcopenia increases complications and mortality after hip arthroplasty. The psoas muscle–L3 vertebra ratio (PML3) is a sarcopenia marker. This study evaluated PML3’s role in predicting postoperative outcomes and guiding surgical decision-making. **Methods**: A retrospective study was conducted at a single centre between January 2021 and December 2024. PML3 was measured on computed tomography (CT) at the L3 vertebra level for patients with displaced FNFs, comparing postoperative outcomes between HA and THA cohorts. **Results**: Eighty-three patients (fifty-seven female, twenty-six male) were analysed. Forty-three underwent THA, and forty underwent HA. Postoperative complications were higher in HA patients (48% vs. 21%, *p* = 0.019), with lower 30-day survival (90% vs. 98%). Median PML3 in the HA group was 0.70 mm^2^ (IQR: 0.47–1.47), lower than in the THA group (1.34 mm^2^, IQR: 1.00–1.78, *p* = 0.002). However, PML3 values for patients that suffered complications (irrespective of surgical decision) were essentially the same; HA, 0.57 mm^2^ (IQR: 0.43–1.83); THA 0.56 mm^2^ (IQR: 0.41–1.05, *p* = 0.847). ROC analysis showed PML3 as an acceptable predictor of postoperative complications, with an AUC of 0.71. **Conclusions**: Lower PML3 values correlate with higher postoperative complications and mortality following THA or HA for displaced FNFs, confirming its role as a prognostic marker. Some THA complications in low-PML3 patients might have been avoided by selecting less invasive HA, suggesting THA should be reserved for those with greater muscle reserves.

## 1. Introduction

Femoral neck fractures (FNFs) are common in older adults, leading to significant morbidity and disability, and requiring rapid surgical treatment [1,2,3]. Displaced FNFs are treated with either a total hip arthroplasty (THA) or hemiarthroplasty (HA), with the important choice as to which being essentially at the discretion of the surgical teams.

National Institute for Health and Care Excellence (NICE) guidelines recommend THA for patients with a displaced intracapsular hip fracture who (i) were able to walk independently out of doors with no more than the use of a stick, (ii) do not have a condition or comorbidity that makes the procedure unsuitable for them (iii) are expected to be able to carry out daily living activities independently beyond 2 years [4]. Selecting the wrong operative method can lead to devastating complications. Frail patients with low physiological reserve qualified for THA are exposed to a longer operative times and have an increased risk of postoperative blood transfusions and complications [5,6]. Further, patients who are not able to conform to postoperative rehabilitation protocols are at an increased risk of prosthesis dislocation and periprosthetic fractures due to falls and poor rehabilitation [7]. Equally, HA patients who lead an active lifestyle report poorer hip functional score outcomes and are more susceptible to acetabular erosion and periprosthetic fractures [8,9].

Proximal femur fractures are intrinsically linked to sarcopenia and frailty syndrome. In our recent systematic review, we demonstrated that sarcopenic orthopaedic patients have increased morbidity and mortality rates, but also increased risk of hospital infections and decreased functional outcomes following surgery [10]. Sarcopenia is characterised by the loss of skeletal muscle mass, strength, and function. Ideally, diagnosis is based on measures of both low muscle strength and mass [11]; however, this is particularly challenging in this cohort, where strength measurements are often inaccurate and gait speed tests are not possible due to the nature of the injury. There is no consensus on the best alternatives to diagnose sarcopenia in orthopaedic patients, and molecular biomarker studies are still limited [12,13,14]. Despite this, imaging-based muscle mass measurements can be useful. The psoas muscle–L3 vertebra ratio (PML3) is a marker of muscle mass obtained from abdominal computed tomography scans. Reduced PML3 levels are linked to sarcopenia and have also been identified as a potential preoperative predictive tool for outcomes in abdominal surgery [15,16]. Previous research has compared PML3 to PMI (psoas major area) normalised to patient’s height and to PBSA (psoas major area normalised to patient’s body surface area) showing that PML3 is the most accurate predictor of postoperative complications and mortality [17,18]. This study aimed to evaluate the use of PML3 as a predictor of postoperative complications in patients undergoing THA and HA for displaced FNFs and to explore its potential use as a tool for informing clinical decision-making.

## 2. Materials and Methods

Retrospective observational data collection was performed to assess PML3 scores in patients undergoing HA and THA and to determine if those scores were predictive of postoperative complications. All data were collected from an orthopaedic tertiary referral centre between January 2021 and December 2024. Patients were identified in the hospital database through the ICD-10 codes. Institutional approval was granted prior to data collection.

Patients were included if they sustained a displaced FNF (AO/OTA 31B1.3, 31B2, 31B3) and were qualified for either HA or THA. Patients were assessed at the time of admission to the orthopaedic ward by senior consultants. Patients who were expected to be able to carry out daily living activities independently beyond 2 years and fully independently mobilise were qualified for THA, in keeping with NICE guidelines [4]. HA was offered to patients who were expected to not mobilise independently after surgery. Patients with nondisplaced FNFs undergoing internal fixation were excluded from analysis. Patients with other concomitant fractures or trauma were excluded. Patients who were disqualified from surgery or died prior to surgery were also excluded. Data collection was performed by one researcher (F.B.) and eligibility criteria was determined using a screening log.

**Figure 1:**
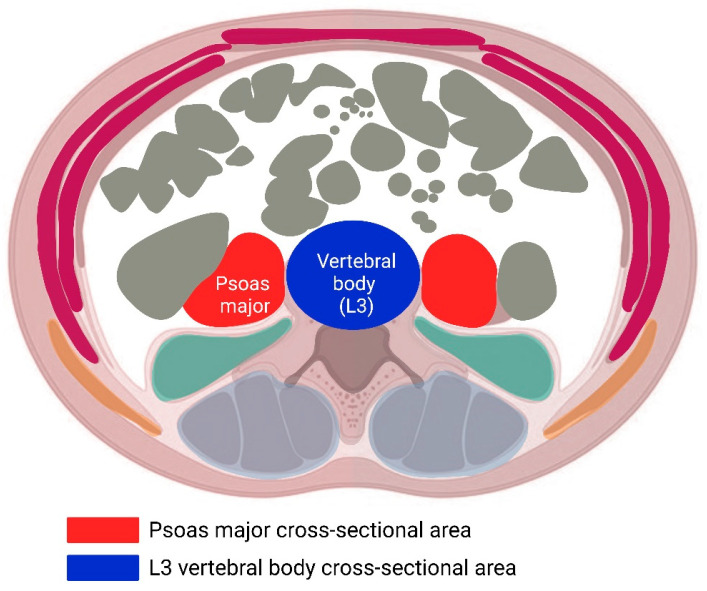
Schematic representation of PML3 measurement based on psoas major assessment on an abdominal CT scan at the L3 level, normalised to the L3 vertebral body (psoas muscle-to-L3 ratio, PML3). Created in BioRender. Dziedzic, A. (2025) https://BioRender.com/g64e900 (accessed on 30 January 2025).

The psoas area-to-third lumbar vertebra (PML3) ratio was used to assess low muscle mass as an indicator of sarcopenia, and was measured by a single orthopaedic surgeon. Patients who had a computed tomography (CT) scan within one year prior to the operation were included in the study (Infinitt, Phillipsburg, NJ, USA). Patients were excluded if their preoperative CT scan was older than one year. PML3 ratio was measured as described in previously published research [17,18]. PML3 was calculated for each patient on preoperative CT imaging at the level of the inferior endplate of the L3 vertebra (Figure 1). Surface areas of both psoas muscles were measured and the sum of both areas was divided by the measured surface area of the L3 vertebral body. The final score was measured in millimetres squared (mm^2^).

Patient demographics, including sex, age and associated comorbidities were collected. Postoperative outcomes including 30-day survival, postoperative hospital acquired infections, surgical site infection rates, length of hospital stay and the need to transfuse was collected. Patients with haemoglobin levels < 8.5 g/dL were given transfusions, and associated symptoms such as lethargy, weakness, and tiredness, as well as likely secondary effects, including syncope, shortness of breath, and reduced exercise tolerance. Patients with haemoglobin levels of <8.0 g/dL were all transfused. All patients were transfused with 2 units of packed red cells. The severity of complications was also graded as per the Clavien–Dindo classification. Grade I represented minor complications needing no specific treatment, while grade II involved complications managed pharmacologically or non-surgically. Grade III denotes complications requiring interventions. Life-threatening complications are classified as IVs (without ICU admission) and IVb (with ICU admission). Finally, grade V signified patient mortality [19]. Patient discharge locations were recorded as a measure of independence following surgery. The primary objective was to assess the differences in PML3 scores between patients who had HA and THA and determine if those scores were predictive of postoperative complications in both groups of patients. All data were obtained from the protected online hospital records.

Statistical analyses were performed using Statistica v. 14.1.0.4 (TIBCO Software Inc., Palo Alto, CA, USA) and SigmaPlot 11.0 (Systat Software Inc., San Jose, CA, USA) with results visualised through GraphPad Prism v.8.0.1. (GraphPad Software Inc., San Diego, CA, USA). The Figure was generated using BioRender (Toronto, ON, Canada). The assessment of normality was performed using the Shapiro–Wilk test. Data following a normal distribution were analysed using Student’s *t*-test, while data not conforming to normal distribution were evaluated using the Mann–Whitney U test. A two-proportion Z-test was used to compare proportions between two independent groups. The association between the studied groups and the occurrence of postoperative complications was assessed using a chi-square test with Fisher’s exact test, based on a contingency table. Additionally, an odds ratio (OR) with a 95% confidence interval (CI) was calculated to quantify the strength of the observed association. The diagnostic performance of the predictor was assessed using the Receiver Operating Characteristic (ROC) curve and the Area Under the Curve (AUC) to evaluate its discriminative ability. The optimal cutoff was chosen using Youden’s J statistic, balancing sensitivity and specificity. Statistical significance was defined as *p* < 0.05, with significant results (reported to three decimal places) presented in bold; results not meeting this threshold were classified as non-significant (n.s.).

Two tasks were performed using AI tools (ChatGPT-4.0, OpenAI, 2024): (1) imputing missing demographic data (length of stay in the hospital) for one patient using the Random Forest model, a machine learning algorithm that relies on an ensemble of decision trees. This model considered key variables such as sex, age, BMI, type of surgery, and the presence of complications. And (2) generating the ROC curve, with the cut-off point clearly marked using Python-based visualisation tools (v. 3.10, Python Software Foundation, Beaverton, OR, USA). The resulting ROC curve is identical to the one generated by the statistical software.

This research received no specific grant from any funding agency in the public, commercial, or not-for-profit sectors.

## 3. Results

### 3.1. Demographics

**Figure 2:**
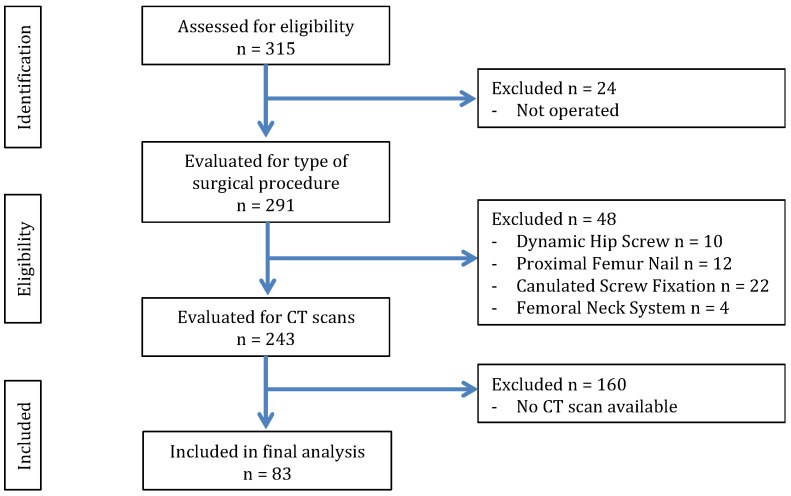
Flow diagram showing patient exclusion process in the study.

A total of 315 patient records with FNFs were reviewed. Of these, 24 were excluded for not undergoing surgery, and an additional 48 were treated for nondisplaced FNFs. Among the remaining cases, 83 patients had a CT scan, meeting the inclusion criteria for the study (Figure 2). In total, 83 patients were included in the study of which 26 were males and 57 were females (Table 1). In total 43 patients underwent THA with mean age of 69 (IQR: 64–74) and 40 patients who underwent HA with a mean age of 84 (IQR: 75–90, *p* < 0.0001). Mean BMI in THA was 25.72 (IQR: 23.44–29.37) and in HA was 25.13 (IQR: 20.18–27.11, *p* = 0.126). Patients undergoing THA had a significantly shorter mean length of hospital stay (LOS) compared to HA patients (*p* = 0.036). The Z-test for proportions revealed that hypertension (72.5% vs. 51%), cardiovascular disease (47.5% vs. 21%), and active cancer (17.5% vs. 5%) were significantly more prevalent in the HA group compared to the THA group. No significant difference was observed for diabetes (22.5% vs. 21%) between the two groups.

### 3.2. Postoperative Outcomes

Postoperative complication rates were more than twice as high in the HA group (48%, 19/40) compared to the THA group (21%, 9/43), with a statistically significant difference (OR 3.42; 95% CI 1.24–8.62; *p* = 0.019) (Table 2). The median of PML3 score in the HA group was 0.70 mm^2^ (IQR: 0.47–1.47) and was significantly lower compared to the THA group’s 1.34 mm^2^ (IQR: 1.00–1.78, *p* = 0.002). However, in those with complications, PML3 was comparable between THA 0.56 mm^2^ (IQR: 0.41–1.05) and HA groups 0.57 mm^2^ (IQR: 0.43–1.83, *p* = 0.847) (Table 2). Further, in the THA group there was a noticeable difference in the PML3 score between the THA group mean and those patients that went on to have complications (1.34 vs. 0.56 mm^2^).

Although the 30-day survival was numerically lower in the HA group compared to the THA group (90% vs. 98%, respectively), this difference was not statistically significant (*p* = 0.191) (Table 2). Other major postoperative complications such as bleeding, cardiovascular incidents, pneumonia, urinary tract infections, and wound infection were less frequent in the THA group in comparison to HA group (Table 2). Reoperation rates were comparable between the THA and HA groups, with both at 5%. The analysis showed that patients in the HA group had a higher risk of complications compared to the THA group, with a statistically significant increase in bleeding risk (OR = 4.44, 95% CI = 1.12–17.57, *p* = 0.034). For the remaining complications, the HA group showed higher OR values, but none reached statistical significance.

In patients who experienced complications, the demographic and clinical profiles differed between the THA and HA groups. Among patients with complications in the THA group, the mean age was 67.44 years (SD 5.96), with comorbidity rates as follows: hypertension, 11.63%; cardiovascular disease, 4.65%; diabetes, 6.98%; pulmonary disease, 0%; dementia, 0%; and cancer, 2.33%. In contrast, patients in the HA group with complications had a mean age of 81.89 years (SD 11.8), with higher comorbidity rates, as follows: hypertension, 37.50%; cardiovascular disease, 27.50%; diabetes, 15%; pulmonary disease, 2.5%; dementia, 0%; and cancer, 7.5%. These findings indicate that patients with complications in the HA group tended to be older and had more comorbidities compared to those in the THA group.

Analysis of Clavien–Dindo complication grades revealed that no complications (grade 0) occurred more frequently in the THA cohort (70% vs. 40%, *p* = 0.008), while severe complications (grade V, mortality) were higher in the HA group (2% vs. 10%). There was a much higher proportion of patient discharge home in the THA group (93%) compared with the HA group (47%) (Table 3). In the THA cohort, the remaining patients discharged were sent home with social care support. In HA, 31% were discharged home with social care support, 6% to a rehabilitation unit and 17% to a nursing home.

**Figure 3:**
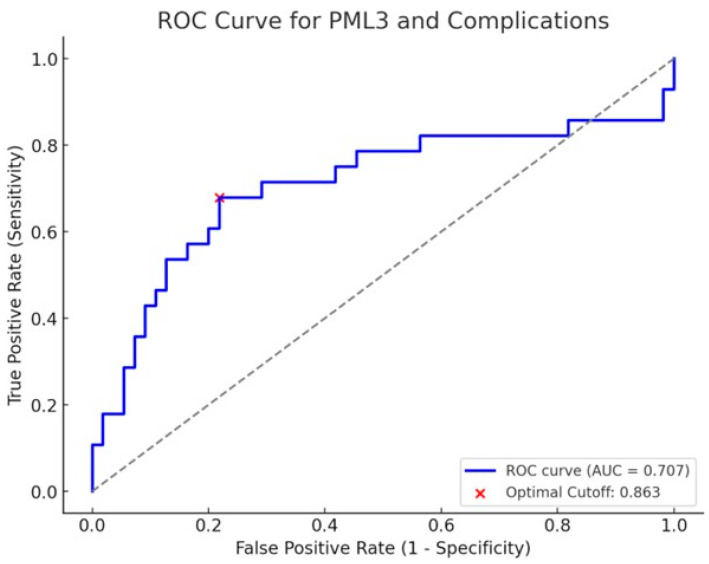
ROC curve of PML3. Source: OpenAI, generated using ChatGPT-4o (accessed 30 January 2025).

ROC analysis for PML3 as a predictor of postoperative complications yielded an AUC of 0.71, indicating a moderate diagnostic ability. The optimal cut-off value was determined to be PML3 ≤ 0.86, maximising sensitivity (68%) and specificity (79%). An AUC of this magnitude suggests that PML3 can distinguish between patients with and without complications better than chance, but its performance remains suboptimal for use as a standalone diagnostic tool. The optimal cut-off point was determined to be PML3 ≤ 0.86, based on the Youden Index, which balances sensitivity and specificity. At this threshold, the sensitivity was 68%, and the specificity was 79%. These values imply that the test correctly identifies 68% of patients who experience complications (true positives), while 79% of patients without complications are accurately classified as such (true negatives). The sensitivity of 68% reflects a moderate true positive rate, indicating that a proportion of complication cases may remain undetected (false negatives). Conversely, the specificity of 79% demonstrates relatively good capability in ruling out complications, though a small fraction of patients may be incorrectly flagged as at-risk (false positives). While these diagnostic metrics support the potential clinical utility of PML3, particularly in stratifying risk, caution is warranted. The moderate AUC and imperfect sensitivity/specificity suggest that PML3 should not be relied upon in isolation but may be valuable as part of a multivariable model or in conjunction with other clinical indicators to enhance predictive accuracy. In summary, PML3 shows promise as a biomarker for postoperative complication risk, with a reasonable balance between sensitivity and specificity at the chosen threshold. Further validation in independent cohorts and exploration within multivariate frameworks is recommended to confirm its predictive value and clinical applicability (Figure 3, Table 4).

## 4. Discussion

This retrospective observational study assessed the utility of PML3 as a predictor of postoperative outcomes in patients undergoing HA or THA for displaced FNFs. While the ‘fitter’ patients were purposefully selected by the clinical teams to receive THA and the ‘less fit’ HA, our findings demonstrate a significant association between lower PML3 values and increased postoperative complications and decreased 30-day survival irrespective of surgical decision. Patients who experienced complications following THA had significantly lower PML3 scores compared to the average for the entire THA group, suggesting that, in hindsight, these individuals may have been inappropriately selected for the physiologically more demanding procedure. These results align with the established link between sarcopenia and poor outcomes following hip arthroplasty, as well as other orthopaedic interventions, with PML3 serving as a readily available surrogate marker for muscle mass [10,12].

The diagnostic performance of PML3 as a predictor of postoperative complications was assessed using the ROC curve and the AUC. The ROC analysis was performed to evaluate the discriminative ability of PML3, with an AUC value of 0.71, indicating an acceptable predictive capability. Previous research, similarly, has shown the PML3 ratio as a useful prognostic indicator for the prediction of mortality in the setting of emergency laparotomy, in addition to it being a superior mortality predictor over the P-POSSUM score (Physiological and Operative Severity Score for the enumeration of Mortality and Morbidity) [17].

Patients with complications who underwent THA had significantly lower PML3 scores (0.56 mm^2^) than the average PML3 score for the entire THA group (1.34 mm^2^) and even lower than the average for the HA group (0.70 mm^2^). This raises important questions about patient selection for THA. The results suggest a potential mismatch between patient frailty, as indicated by PML3, and the surgical approach chosen. While these data do not suggest that these patients were wrongly qualified for THA, it strongly implies that low muscle mass, reflected in a low PML3 score, might be a significant factor predicting poorer outcomes in patients undergoing THA. This suggests that, for some patients considered for THA, a clinician’s assessment of frailty could be supplemented with a PML3 score evaluation to objectively confirm their frailty. This may help guide surgical decision-making, potentially leading to the selection of a less demanding HA procedure when appropriate.

Previous research suggests that significant discrepancies exist between clinician assessment and patient self-assessment of functional capacity [20]. In our study, patients were qualified for THA based on the NICE guidelines [4]. The preoperative assessment relied on the surgeon’s subjective judgement, potentially overestimating a patient’s ability to tolerate THA and increasing complications in those with undetected frailty. The sensitivity of existing frailty scores in detecting clinically significant levels of frailty is limited and the objective data provided by PML3 could serve as a valuable adjunct to clinical judgement, helping to identify patients at higher risk of complications and guide more accurate surgical decision-making [21].

The higher complication rate (48%) and lower 30-day survival (90%) in the HA group compared to the THA group (21% and 98%, respectively) suggests that, on average, patients qualified for the HA in our study overall were more frail. Overall, patient-surgery selection generally adhered to the NICE criteria. However, PML3 could serve as a valuable tool to identify borderline cases where clinicians may be uncertain about whether a patient best qualifies for THA or HA. Research suggests that early complications are higher after THA than HA; however, a key study excluded dementia and malignancy, which were more common in our HA cohort [22]. Voskuijl et al. (2014) found that, when controlling for demographics and comorbidities, HA patients had a 40% higher risk of adverse events than THA patients [23]. Differences in surgical qualification criteria may explain these findings.

There are several limitations of this study. The sample size of this study is limited; however, this is because most studies rely on abdominal CT scans, which are not frequently performed in orthopaedic patients, which reflects a broader challenge in orthopaedic sarcopenia research. Most patients included in our study had undergone CT scans for malignancy screening or lumbar spine fracture assessment within one year prior to surgery. In other units and healthcare systems, CT scans may not be as readily available. The PML3 parameter was measured by a single surgeon, potentially introducing subjective bias into the measurement process, and future research should utilise multiple blinded assessors to improve the reliability of the measured PML3 scores. Finally, the 30-day follow-up may not capture long-term outcomes such as acetabular erosion or periprosthetic fractures [24,25]. As these complications often develop over time, future studies with extended follow-ups are needed to better assess the long-term impact of surgical decisions and patient recovery

Future research should focus on replicating these findings in a multicentre setting with larger, more diverse cohorts to enhance the generalizability and robustness of the results. Conducting studies across multiple institutions will help account for variations in patient demographics and clinical practises. Furthermore, multicentre studies can evaluate the accessibility of abdominal CT scans for orthopaedic patients and assess the feasibility of obtaining reliable muscle mass measurements across different clinical settings.

## 5. Conclusions

In conclusion, although this study is limited, the results suggest a potential mismatch between patient frailty, as indicated by PML3, and the surgical approach chosen to treat displaced FNFs. Current guidelines for THA vs. HA may inadequately assess frailty, relying on bedside functional assessments of patients, which may not fully objectively capture the frailty and sarcopenia impact. PML3 offers a more objective measure of muscle mass, potentially identifying patients with sarcopenia and at increased risk of complications. If an abdominal CT scan from the past year is available, clinicians should consider using PML3 measurements to assess the risk of complications, which may aid in decision-making between selecting THA and HA in borderline cases. However, additional research is needed to confirm these findings and establish specific PML3 threshold values that would influence surgical decisions.

## Figures and Tables

**Table 1:** Patient demographics and comorbidity rates.

	Total Hip Arthroplasty (THA) (n = 43)	Hemiarthroplasty (HA) (n = 40)	*p*-Value (Z-Score) ^a^
Gender [Female—F; Male—M]	F = 25 (58%) M = 18 (42%)	F = 32 (80%) M = 8 (20%)	***p* = 0.032 ^a^** **(−2.15)**
Age (median with IQR) [years]	69 (64–74)	84 (75–90)	***p* < 0.0001 ^b^**
Body mass index (BMI) (median with IQR) [kg/m^2^]	25.72 (23.44–29.37)	25.13 (20.18–27.11)	*p* = 0.126 ^b^ (n.s.)
Length of hospital stay (LOS) (median with IQR) [days]	10 (7–12)	12.5 (9–16)	***p* = 0.036 ^b^**
Smoking habit	11 (25.5%)	5 (12.5%)	*p* = 0.131 ^b^ (n.s.)
Comorbidities:			
Hypertension	22 (51%)	29 (72.5%)	***p* = 0.046 ^a^** **(−1.99)**
Cardiovascular disease	9 (21%)	19 (47.5%)	***p* = 0.011 ^a^** **(−2.56)**
Diabetes	11 (21%)	9 (22.5%)	*p* = 0.743 ^a^ (0.33)
Pulmonary disease	0	2 (5%)	-
Dementia	0	3 (7.5%)	-
Active cancer	2 (5%)	7 (17.5%)	*p* = 0.060 ^a^ (−1.88)

^a^ The difference was tested using a two-sided z-test for proportions. ^b^ Non-parametric Mann–Whitney U test was used to assess the difference between the groups. (n.s.)—non-significant. Bolded values represent results that reached statistical significance *p* < 0.05.

**Table 2:** PML3 scores, postoperative outcomes, and Clavien–Dindo classification.

	Total Hip Arthroplasty (THA) (n = 43)	Hemiarthroplasty (HA) (n = 40)	*p*-Value OR (95% CI)
PML3 score (median with IQR) [mm^2^]	1.34 (1.00–1.78)	0.70 (0.47–1.47)	***p* = 0.002 ^a^**
PML3 score in patients with complications (median with IQR) [mm^2^]	0.56 (0.41–1.05)	0.57 (0.43–1.83)	*p* = 0.847 ^a^ (n.s.)
Postoperative complications in overall	9 (21%)	19 (48%)	***p* = 0.019 ^b^** 3.42 (1.24–8.62)
30-day survival	42 (98%)	36 (90%)	*p* = 0.191 ^b^ 0.21 (0.02–1.42)
Required Transfusion	3 (7%)	10 (25%)	***p* = 0.034 ^b^** 4.44 (1.12–17.57)
Pneumonia	1 (2%)	4 (10%)	*p* = 0.191 ^b^ 4.67 (0.50–43.67)
Urinary tract infection	1 (2%)	3 (7.5%)	*p* = 0.348 ^b^ 3.41 (0.34–34.17)
Cardiovascular incidents	3 (7%)	5 (12.5%)	*p* = 0.473 ^b^ 1.90 (0.42–8.55)
Wound infection	1 (2%)	2 (5%)	*p* = 0.607 ^b^ 2.21 (0.19–25.37)
Gastrointestinal issues	-	5 (12.5%)	-
Reoperations	2 (5%)	2 (5%)	-
Clavien–Dindo grade:			
0	30 (70%)	16 (40%)	***p* = 0.008 ^b^** **0.29 (0.12–0.72)**
I	4 (9%)	5 (12.5%)	*p* = 0.732 ^b^ 1.39 (0.35–5.60)
II	5 (12%)	10 (25%)	*p* = 0.155 ^b^ 2.53 (0.78–8.21)
III	2 (5%)	4 (10%)	*p* = 0.422 ^b^ 2.28 (0.39–13.18)
IV	1 (2%)	-	-
V	1 (2%)	4 (10%)	*p* = 0.191 ^b^ 4.67 (0.50–43.67)

^a^ Non-parametric Mann–Whitney U test was used to assess the difference between the groups. ^b^ chi-square test with Fisher’s exact test, based on a 2 × 2 contingency table with an odds ratio (OR) with a 95% confidence interval (CI) calculated. Bolded values represent results that reached statistical significance *p* < 0.05.

**Table 3:** Patient discharge location outcomes of THA and HA patients.

Discharge Location	Total Hip Arthroplasty (THA) (n = 42)	Hemiarthroplasty (HA) (n = 36)	*p*-Value (Z-Score)
Home	39 (93%)	17 (47%)	***p* < 0.001** **(4.46)**
Home with social care support	3 (7%)	11 (31%)	***p* = 0.007** **(−2.69)**
Rehabilitation unit	-	2 (6%)	*p* = 0.122 (−1.55)
Nursing home	-	6 (17%)	***p* = 0.006** **(−2.75)**

Bolded values represent results that reached statistical significance *p* < 0.05.

**Table 4:** Metrics of ROC analysis for PML3 as a predictor of postoperative complications.

Metrics	Value
Area Under the Curve (AUC)	0.71
Optimal Cut-off (PML3 [mm^2^])	0.86
Sensitivity (%)	68%
Specificity (%)	79%

## Data Availability

The data presented in this study are available on request from the corresponding author due to patient confidentiality and ethical considerations, as they contain sensitive medical information that cannot be publicly shared.
